# An Experimental Inquiry into the Pathology and Treatment of Asphyxia

**Published:** 1846-01

**Authors:** 


### ART. XXII.

*An experimental Inquiry into the Pathology and Treatment of Asphyxia.* By JOHN E. ERICHSEN.—*Edinburgh*, 1845. pp. 56.

THE author of the present treatise was appointed by the general committee of the British Association for the Advancement of Science, at the meeting held at Manchester in the year 1842, to form, in conjunction with Dr. Sharpey, a commission to make an experimental inquiry into the subject of asphyxia. Professor Sharpey shortly afterwards retired from the commission, and our author continued to prosecute his inquiry alone. His report, which was read at the fourteenth meeting of the British Association, and for which the Royal Humane Society awarded the Fothergillian gold medal, is the work before us.

Mr. Erichsen first considers his subject with reference to its pathology, and then enters into the question of the treatment of asphyxia.

In the first part of the subject, the pathology of asphyxia, Mr. Erichsen examines experimentally the merits of the three leading opinions each of which has, in turn, been received as the fundamental *fact* of asphyxia:

"1st, That the circulation ceases in consequence of the arrest of the respiratory movements; 2d, in consequence of want of power in the heart; 3d, in consequence of an obstruction in the passage of blood through the lungs" (p. 2.)

Each of these opinions has received the investigation of careful, and, in our opinion, in the main, conclusive experiments; though, as we have often had occasion to express ourselves before, so great do we consider the violence done to the system in these experiments on living animals, sufficient of themselves to cause death, that we cannot accept all their results without some reservation.

With regard to the first and second of the above views of the leading cause of the phenomena of asphyxia, as the conclusions of Mr. Erichsen are in unison with those of Dr. Kay, now, we believe, so generally received,—and for a concise and clear expression of which we may refer to a book in every one's hands, the excellent lectures of Dr. Watson (vol. i, p. 45)—we do not deem it needful to follow him through his reasonings and experiments, but shall satisfy ourselves with expressing his conclusions, only adding on our own part, that we consider them borne out by his experiments. We give them in his own words:

"1st. That although the persistence of the respiratory movements has some influence in maintaining the circulation through the lungs, yet that their arrest is not by any means the sole cause of the cessation of the circulation.

"2d. That a diminution in the force and frequency of the contractions of the heart, consequent upon the altered quality and lessened quantity of the blood circulating through its muscular substance, is one of the principal causes of the cessation of the circulation in asphyxia, as is evident from the fact, that, when the force of the heart's contractions is maintained by a supply of arterial blood to its muscular substance, it is enabled to propel black blood through a collapsed lung." (p. 31.)

It is, however, in the investigation of the third opinion, the obstruction of the circulation through the lungs, that Mr. Erichsen finds the main cause of the arrest of the circulation. As on this point, however, we find him somewhat at issue with Dr. Kay, whose opinions on this, as on the other heads, have, we believe, received general assent, we here propose to follow him, in some measure, into the details of his reasoning and experiments. While Dr. Kay holds "that the main cause," we quote the clear and concise account of Dr. Watson,* "of the failure of the circulation seems to be the difficulty with which the non-arterialized blood finds its way though the capillaries of the lungs," Mr. Erichsen, on the other hand, is of opinion that the obstruction is in the passage through—not the capillaries but—the smaller ramifications of the pulmonary veins, believing "that the venous blood which circulates in asphyxia may act as a stimulus to the smaller branches of the arterial system, [by analogy, those of the pulmonary *veins*,] exciting the contractility of these vessels, and that thus the obstruction in the circulation may be occasioned." (p. 26.) Mr. Erichsen dismisses the claim of the action of the capillaries in producing this result, by arguing, as "stated by Dr. Alison, that the stagnation cannot arise from the stimulus of the venous blood, being insufficient to excite the capillaries, as it has not been proved that these vessels are irritable, nor that they possess coats." (p. 26.) But, to proceed from the negative

* Lectures on the Practice of Medicine, vol. i, p. 45.

to the positive side of the argument, our author observes, "we find besides, that every part of the body [he has made an exception in the previous page in favour (?) of the capillaries] is provided with a special sensibility, 'which determines,' to use the words of Bichat, 'the nature of its relations with those foreign bodies which may happen to be in contact with it." (p. 27.) Mr. Erichsen then cites the peculiar physiological relations and actions, towards foreign bodies, of the salivary, bilious, and other ducts, and, reasoning from them towards the relations between venous blood and arterial branches, he remarks, "on reasoning from analogy, therefore, we should be disposed to look upon venous blood as a stimulant to the arterial system, that is, as having a tendency to excite the contractility of these vessels."

"But," continues Mr. Erichsen, "we may go a step further, and prove that it actually possesses this power; causing these vessels to contract distinctly, as I have several times observed, on examining under the microscope, the mesentery of rabbits during and immediately after the process of asphyxia. . . . On asphyxiating a young rabbit, a portion of whose mesentery has been conveniently fixed under a powerful microscope, the following phenomena will be observed to ensue. For about a minute after the struggles of the animal have ceased the circulation appears to be going on with its usual rapidity; it then gradually becomes somewhat slower, the arteries contracting in size, containing less blood, and assuming a lighter and more tawny colour than before; whilst the veins become congested, and evidently fuller, assuming, when viewed by transmitted light, a very beautiful crimson hue As the circulation becomes more languid, the arteries continue contracting, and acquire a lighter colour; the diminution in their size and the difference in the quantity of blood contained in them and in the veins, being most marked. The motion of the blood in the capillaries now becomes oscillatory, the whole mass of the blood being at each impulse of the heart slowly propelled forwards, and then moving backwards. . . . Nor have I ever been able to discover any obstruction in the vessels, in consequence of the adhesion of colourless globules to their sides,—a phenomenon that I especially watched for, and which has by several been supposed to occur. The diminution in the diameter of the smaller arteries, and the proportionate difference between these and the neighbouring veins, was most evident, and was such as could leave no doubt on my mind as to the important part that the contractions of these vessels play, in giving rise to an obstruction to the passage of the blood through them in asphyxia; *in which I have no doubt it is the principal if not the sole agent.*" (pp. 28-9.) [The italics are our own.]

To apply these general remarks to the circulation through the lungs, Mr. Erichsen observes:

"The blood having now become perfectly venous, begins to be obstructed in its passage through the ultimate ramifications of the pulmonary veins, and of the arterial system generally, occasioning congestions of the pulmonary artery, of the right cavities of the heart, and of the whole of the venous system; and the quantity sent to the left cavity of the heart becomes materially lessened." (p 30.)

Having thus given our author's opinions and reasonings on this head, we quote his two remaining conclusions, viz.:

"3d. That the obstruction which has been proved to take place in the pulmonary and systemic circulation is due to the venous blood exciting the contractility of the minute divisions of the arteries and pulmonary veins, by acting upon their special sensibility.

"4th. That the cause of the stoppage of the circulation in asphyxia is therefore threefold: depending, first, upon the arrest of the pulmonary movements; second, upon the weakening of the heart's action; and third, upon the obstruction offered to the blood (propelled with diminished force) by the refusal of the pulmonary veins and minute arteries to receive venous blood." (pp. 31-2.)

Before quitting this part of our subject we feel tempted to transcribe a table of the average duration and succession of the various phenomena of asphyxia, as noticed by Mr. Erichsen in his numerous experiments. The animals experimented on were dogs.

| | | Minutes. |
|---|---|---|
| "Voluntary movements cease in . . . . . | | 1¾ |
| Involuntary movements in . . . . . | | 2½ |
| Blood in arteries becomes as black as that in the veins in . | | 1¾ |
| Occasionally as early as . . . . . | | 1¼ |
| Contractions of the ventricles cease in . . . | | 9½ |
| The earliest that I have observed has been in . . | | 6½ |
| The latest in adult animals in . . . . . | | 14 |
| Twitching and irregular movements continue some time longer. | | |
| The left auricle on an average in . . . | | 12 |
| I have seen it in an adult continue to contract till the . | | 37th |
| The right auricle on an average in . . . | | 19½ |
| I have seen it in an adult animal continue to contract till the | | 44th |
| Pulsations continue in the femoral artery on an average for | | 7½ |
| In very young animals the time that the contractility of different parts of the muscular system is maintained is very different. | | |
| In puppies four days old movements continued for . . | | 16 |
| Ventricles continued acting regularly for . . | 1 hour | 57 |
| But twitchings and irregular movements continued for | 3 hours | 4 |
| Auricles continued acting for . . . . | 3 hours | 25." (p.31.) |

Under the head of treatment, Mr. Erichsen submits some interesting considerations. Can the circulation be restored through the lungs after it has become stagnated in that organ? Can the action of the heart be restored after it has ceased pulsating? (p. 41.) Each of these considerations demands some notice, which we shall make as concise as possible.

First, with respect to the renewal of the circulation through the lungs, after it had actually ceased in those parts. Mr. Erichsen gives us the detail of an experiment,—and he informs us he repeated it many times with the same result,—in which, though artificial respiration was not applied till 11½ minutes after the auricles had finally ceased from their irregular movements, and the left side of the heart was emptied, and as much as 37½ minutes after the ventricles had ceased to contract, yet that after commencing artificial respiration, the blood in the pulmonary veins became arterial, and blood returned to the left side of the heart, insomuch that after four minutes the auricles became of a more florid hue; and at the 27½th minute of artificial respiration, he informs us, "on puncturing the left auricle at the point of junction with the pulmonary veins, a very large quantity of perfectly bright arterial blood issued in a small jet a line or two in height. The insufflation being continued, perfectly florid blood continued to well out each time the lung was distended." (p. 43.) Still, however, there was no renewal of the systemic circulation. We think with our author he has made out his case, that his experiments clearly prove "the possibility of re-establishment of the circulation through the lungs after the heart's action has entirely ceased." (p. 43.)

We now come to consider the second question proposed, viz., the possibility of restoring the heart's action after *it* had ceased; but before expressing our conclusion on this point, we must discuss as preliminary to it, a question on which our author appears to have come to an erroneous conclusion; it is this,—how long after submersion does the heart continue to pulsate? This may vary; syncope may be induced from mental terror, and the circulation, and the consequent changes of the blood being thus considerably retarded, the action of the heart, though extremely feeble, as of necessity it must be in this condition, may not yet be entirely arrested for a much longer period than in other cases, when the violent struggles not only force out the air contained in the lungs, but by quickening the circulation, much sooner reduce the blood to the venous character, and thus secondarily must hasten the period at which the heart ceases to act: the quantity of air which happened to be in the lungs at the time, may also vary; but making allowance for these variations, our author is disposed to consider five minutes as the average period after submersion at which the heart ceases to beat. We confess we think this estimate rather low, when we consider that with all the violence of the operation of exposing the heart, and the unfavorable circumstances for the continuation of organic life, that yet in the dogs he has experimented upon, Mr. Erichsen gives $9\frac{1}{2}$ minutes as the average period at which ventricular action ceases: nor do we think that by any means Mr. Erichsen's facts warrant his conclusions. The facts are mostly on the authority of Mr. Woolley, who has had ample opportunity of making correct observations on this subject; they are these, — that, as a general rule, no person recovers who has been much more than four minutes submerged, and that the vast majority recover who can be brought from the water under that period; and hence Mr. Erichsen concludes, "I should suppose that contractions of the ventricles have most generally ceased before 5 minutes have elapsed after complete submersion." (p. 34.) This conclusion, however, is on the assumption that air is restored to the lungs, and arterial blood to the heart, within *a few seconds* after removal from the water, whereas we hold, the calculation should not be the period of actual submersion, but the average period between the commencement of complete submersion and that at which the first evidences of convulsive respiration have been evinced: in the recovery of *extreme cases,* this period would fall *within* the minimum of the continuance of the pulsation of the left ventricle after asphyxia had commenced; a conclusion we think fully borne out in a quotation which Mr. Erichsen himself brings forward in refutation of a similar error with respect to a similar calculation in Dr. Roesler's experiments on rabbits.*

"'It may be said .... that an obscure respiration might have been going on' from the time the asphyxiating cause was resumed, 'but every one who is

* Edin. Med. and Surg. Journal, vol. xxiii.

materially familiar with the phenomena of suspended animation, must know that this was impossible. After an animal has been asphyxiated so long that its breathing has ceased, the first efforts to renew respiration are never obscure; but they are convulsive gasps attended with spasm of the whole body; such as no one could possibly fail to notice, who was occupied with preparations to resuscitate it.'" (pp. 44-5.) *

So far from the phenomena of asphyxia beginning to cease the instant the body is recovered from the water, the symptoms may be, and probably are yet increasing for some time, while nevertheless means are successfully proceeding towards recovery; for until air actually reach the lungs, and arterialized blood return to the left side of the heart, all the preliminaries for that end cannot prevent the pulsations becoming more and more distinct.

From the conclusion then of our author, that in the human subject the heart's action ceases within 5 minutes after submersion, we certainly must dissent; but we do not despise the facts and observations of Mr. Woolley on which it is founded. They are worth just their average result and no more.

Having considered this question, we may now fairly return to that from which we started, viz., the heart having ceased to pulsate, can its action be restored?

Our author details many experiments on this head, the results of which are briefly as follows: that although artificial respiration with common air will restore the circulation through the lungs, and cause florid blood to return to the heart, yet that it will not restore the pulsations of the ventricles, these having once ceased; but that although the inflation of common air be insufficient for that purpose, yet when oxygen was substituted, the result was perfectly successful in many cases—only however in those in which its use was adopted very quickly afterwards.

"There can be little doubt," observes Mr. Erichsen, "but that the revival of the contractions of the heart in these cases was due to the very rapid arterialization of the blood in the lungs, and its passage into the left side and tissue of the heart, before the irritability of that organ had become so far extinct that it was unable to contract on the application of its usual stimulus; and that the reason why, by the inflation of the lungs with common air, it is very difficult, if not impossible, to excite perfect contractions of the ventricles, although tremulous movements in them, and proper action of the auricles may be set up, is, that blood does not become arterialized soon enough to reach the left ventricle, and to permeate the tissue of the heart in sufficient quantity before that organ has lost its power of contracting on the application of this stimulus." (p. 52.)

From the results of his experiments we need hardly remark that Mr. Erichsen places artificial respiration with oxygen gas as a most important

* An instance of the convulsive movement attendant on returning respiration we had recently an opportunity of observing so low in the animal kingdom as the class *insecta*. We were rearing a larva of the puss moth, *ceruria visculia*, which we had left feeding on a twig of poplar in a glass of water. On entering our study we found our caterpillar drowned in the glass. We wiped the water from the spiracles on both sides, and held him on the warm hand. After a few minutes a convulsive movement took place in the posterior articulation, with violent throbbing of the corresponding portion of the dorsal artery, very different from the tranquil pulsation which usually exists. This kind of action continued to take place upwards in each consecutive articulation, and our pet was soon restored to his usual functions.

agent in restoring animation. In his mode of applying this remedy, we do not observe any peculiarity worthy of notice, except the advantage of using it warm—for instance, about 98°. With respect to the other means noticed by Mr. Erichsen, we observe nothing different from those recommended in the several excellent articles which our now numerous cyclopædias contain, except that with regard to the warm bath, he lays great stress on its temperature, being in some proportion regulated by the heat of the water in which the patient had been immersed. He observes that while 98° or 100°—the degree recommended by the Royal Humane Society—may be proper in the summer months when the temperature is between 50° and 60°, it is far too high in the winter when it may be little above 32°. "In this case, I should certainly," observes our author, "recommend that the patient be not at first exposed to a greater degree of heat than from 85 to 90 degrees."

We cannot take leave of Mr. Erichsen without testifying the great pleasure we have experienced in the perusal of his Treatise. We think he has done real service towards our knowledge of the subject he has taken in hand, and we should strongly recommend the perusal of his Treatise, as well to those interested in the physiology and pathology of organic life, as to those who may be so circumstanced as to be likely to have the apparently drowned submitted to their care.

---

